# Puumala hantavirus infections in bank vole populations: host and virus dynamics in Central Europe

**DOI:** 10.1186/s12898-017-0118-z

**Published:** 2017-02-28

**Authors:** Daniela Reil, Ulrike M. Rosenfeld, Christian Imholt, Sabrina Schmidt, Rainer G. Ulrich, Jana A. Eccard, Jens Jacob

**Affiliations:** 1 Institute for Plant Protection in Horticulture and Forests, Vertebrate Research, Julius Kühn-Institute, Toppheideweg 88, 48161 Muenster, Germany; 2 Institute for Novel and Emerging Infectious Diseases, Friedrich-Loeffler-Institut, Südufer 10, 17493 Greifswald-Insel Riems, Germany; 3 Institute of Biochemistry and Biology, Animal Ecology, University of Potsdam, Maulbeerallee 1, 14469 Potsdam, Germany

**Keywords:** *Myodes glareolus*, Population dynamics, Puumala virus seroprevalence, Space use, Survival

## Abstract

**Background:**

In Europe, bank voles (*Myodes glareolus*) are widely distributed and can transmit Puumala virus (PUUV) to humans, which causes a mild to moderate form of haemorrhagic fever with renal syndrome, called *nephropathia epidemica*. Uncovering the link between host and virus dynamics can help to prevent human PUUV infections in the future. Bank voles were live trapped three times a year in 2010–2013 in three woodland plots in each of four regions in Germany. Bank vole population density was estimated and blood samples collected to detect PUUV specific antibodies.

**Results:**

We demonstrated that fluctuation of PUUV seroprevalence is dependent not only on multi-annual but also on seasonal dynamics of rodent host abundance. Moreover, PUUV infection might affect host fitness, because seropositive individuals survived better from spring to summer than uninfected bank voles. Individual space use was independent of PUUV infections.

**Conclusions:**

Our study provides robust estimations of relevant patterns and processes of the dynamics of PUUV and its rodent host in Central Europe, which are highly important for the future development of predictive models for human hantavirus infection risk.

## Background

Hantaviruses are zoonotic and emerging pathogens for humans with rodent and other small mammal reservoirs [1]. Currently, hantaviruses are known to occur in two rodent families (Cricetidae and Muridae), in two families of insectivores (Soricidae and Talpidae) and in three families of bats (Rhinolophidae, Nycteridae and Vespertilionidae) [2, 3]. All hantaviruses seem to be associated exclusively with one or a few closely related mammal reservoir species and mostly follow the geographical distribution of the reservoir [2, 4, 5].

In Europe, five rodent-borne hantaviruses (Puumala, Tula, Tatenale, Dobrava-Belgrade, and Seoul virus) and four insectivore-borne hantaviruses (Seewis, Asikkala, Boginia and Nova virus) have been identified [6, 7]. According to current knowledge, only some rodent-borne hantaviruses cause significant disease in humans. Infections occur via inhalation of aerosolised virus particles, which are shed through urine, faeces or saliva. Hantavirus disease, namely haemorrhagic fever with renal syndrome (HFRS), occurs in Eurasia and has been known since the 1930s [8]. Puumala virus (PUUV) is the most important hantavirus in Northern, Central and Western Europe [9, 10]. PUUV-caused disease in humans is termed *nephropatica epidemica* (NE), which is a mild to moderate form of HFRS. The disease is mainly characterized by renal dysfunction or renal failure. Main symptoms are fever, headache, backpain and gastrointestinal symptoms [11]. The much more severe hantavirus cardiopulmonary syndrome is restricted to the Americas [12].

The first detection of PUUV in Germany was in the 1980s during a Belgian military exercise [13]. Since 2001, human hantavirus infections have been notifiable in Germany and a total of 10,403 cases were recorded until December 2016 (Robert Koch Institute: SurvStat@RKI 2.0, https://survstat.rki.de, data status: 31.12.2016). The geographical distribution of human cases in Germany is heterogeneous, with the majority reported from highly endemic regions in southern, western and north-western Germany [5, 14–16]. In outbreak years, the number of human NE cases rises to about 2000 in Germany (SurvStat@RKI 2.0, https://survstat.rki.de, data status: 17.05.2016).

The bank vole (*Myodes glareolus*) is the main, and in Central Europe exclusive, reservoir of the PUUV. The species is distributed all over Germany and in many other European and Asian countries. In Central Europe it inhabits mainly deciduous broad-leaved forests of oak (*Quercus robur*) and beech (*Fagus sylvatica*) [17], but can also occur in hedges, parks, and urban gardens. In temperate forests, food availability for bank voles fluctuates greatly because of seed masting of beech and other tree species, which is triggered by climatic conditions [18–21]. During beech mast years, bank vole populations grow rapidly reaching peak population densities the following year [19, 22–24]. Earlier studies show that in European broad-leaved forests, bank vole populations fluctuate seasonally and multi-annually with population peaks in summer and population outbreaks every 4–7 years [19]. More recent studies demonstrate bank vole outbreaks every 2–3 years [25] due to shortening beech mast intervals [22]. The increase in the number of human PUUV infections is associated with bank vole population peaks [14, 22, 26, 27]. However, the interplay of vole and hantavirus dynamics is much less understood and this may be crucial for hantavirus transmission within host populations ultimately affecting human infection risk. Due to the potentially severe course of the disease and the large number of people affected, understanding the occurrence of human PUUV infections is highly relevant for public health management.

We present the results of a temporally and spatially replicated live trapping study of bank vole populations that link the presence of PUUV specific antibodies in bank voles to their population dynamics, space use and survival. Patterns of bank vole population dynamics are presented for four, and PUUV dynamics for two, geographical regions (PUUV virtually absent from 2 regions) in Germany from 2010 to 2013 covering two vole outbreaks. Results may be relevant for a future development of an early warning system to minimize the risk of hantavirus infections and related adverse effects on public health in Central Europe.

## Methods

### Rodent trapping

Rodent monitoring was conducted in spring, summer, and autumn of 2010–2013 in four study areas in Germany; West (North Rhine-Westphalia, Billerbeck, 51°59.63′N, 7°18.99′E), South (Baden-Wuerttemberg, Weissach, 48°49.88′N, 8°57.71′E), North (Mecklenburg-Western Pomerania, Jeeser, 54°9.75′N, 13°15.55′E), and East (Thuringia, Gotha, 50°57.38′N, 10°39.13′E). Habitats surveyed were mainly beech forests or mixed deciduous forests.

Ugglan multiple live traps (*Grahnab®, Gnosjö, Sweden*) were baited with apple, rodent pellets, rolled oats and peanut curls and wood shavings were provided for insulation. On each plot, a trap grid of 49 (7 × 7) traps with 10 m spacing was set. Traps were pre-baited for 3 days and checked twice a day for 2–3 consecutive days, in the early morning and late afternoon. Thus, each trapping session consisted of multiple (3–7) trapping occasions. In total, three replicate woodland plots per study area (region) (0.2–2.2 km apart) and trapping session were sampled. Every captured vole was marked with a passive integrated transponder (PIT) tag (*LUX*-*IDent s.r.o.®, Lanškroun, Czech Republic*) for individual identification in the scruff. Species and sex were morphologically determined and animals were weighed to the nearest gram with a 50 g-spring scale (*PESOLA AG®, Schindellegi, Switzerland*). Blood samples (20–40 µl) were taken from the facial vein or the retro-orbital sinus and stored at −20 °C until analysis for PUUV-specific antibodies. After processing, animals were released at the point of capture.

### Density estimation

Bank vole populations were assumed to be closed because immigration, emigration, births and mortality were thought to be minimal during a 3 day trapping period. Closed population densities were estimated using program DENSITY 5.0 (http://www.otago.ac.nz/density/) with the spatial detection model SECR (spatially explicit capture-recapture; [28]) using maximum likelihood (ML). If recapture rates or movement were too limited to calculate density estimates, the minimum number alive multiplied by the trapping area (0.36 ha) was used. Density estimates are stated as individuals per hectare throughout.

### Serological PUUV analyses

Blood was analysed in an immunoglobulin G (IgG) enzyme linked immunosorbent assay (ELISA), which uses a yeast-expressed PUUV nucleocapsid protein to test for PUUV antibody presence in the sample. The investigations followed a previously established protocol [29, 30]. Scoring of reactivities (positive, equivocal, negative) followed a previously described decision tree [31]. In further analyses explicit positive or negative results were required, hence equivocal test results were classified as negative. For each state and season mean seroprevalences ± standard deviation were calculated from plots where >4 samples were obtained.

### Space use

The minimum distance moved among traps during a trapping session divided by the number of recaptures was used as a measure of space use to investigate potential cause and consequence of PUUV infection in bank voles [32]. Animals with first captures in the traps at the margin of the trapping grid were excluded to avoid edge effects [33] and to increase the probability of including grid residents. Individuals captured only once were excluded from the analysis because no information about movement was available.

### Seroconversion and survival

Recaptured bank voles, which were tested for PUUV-specific antibodies in a preceding session, could be identified by their individual marking. This allowed studying seroconversion rates and survival in relation to PUUV infection status. Seroconversion is defined as the occurrence of PUUV-specific antibodies in animals found to be seronegative during previous trapping.

## Statistical analyses

### Bank vole population density and PUUV seroprevalence

Annual and seasonal fluctuations in population density as well as PUUV seroprevalence were analysed by univariate analyses of variance (ANOVA) with subsequent post hoc tests (Tukey’s HSD). Population density or PUUV seroprevalence were dependent variables and year, season as well as study area fixed factors. Analyses were performed using R software (*Version 3.2.5., 2016, R Core Team, Vienna, Austria*). Level of significance was α < 5%.

### Correlation of PUUV seroprevalence and bank vole population density

The influence of bank vole population density on PUUV-seroprevalence was statistically analysed using a generalized linear mixed model (GLMM) with binomial distribution and a logit link function (level of significance α < 5%) using R software. PUUV seroprevalence as a proportional response variable (2-vector variable) was generated from the number of PUUV seropositive bank voles and the difference between the number of tested bank voles and the number of PUUV seropositive bank voles (=number of PUUV seronegative bank voles). Population density and the density of the previous session (standardised by z-transformation (z = (x − mean)/sd)), both in interaction with season (factorial variable), were included as covariates. Plot nested in study area and year were included as random factors. Further, an observation-level random effect was added to account for overdispersion [34], which was tested a priori using package ‘blmeco’ and function ‘dispersion_glmer’. The number of observations was N = 58 and the best model was chosen according to Akaike information criterion (AIC). We used function ‘r.squaredGLMM’ from the ‘MuMIn’-package to estimate a pseudo-R^2^ for GLMMs (R^2^ conditional = variance explained by fixed and random factors) [35, 36].

### Space use

Individual differences in space use were investigated under the premise to be either the cause or consequence of PUUV infection. For this the effect of the mean minimum distance moved between recaptures on PUUV infection status (space use = cause) was analysed with a GLMM with binomial distribution and a logit link function (level of significance α < 5%) using R software. PUUV infection status was the dependent variable and ‘mean minimum distance moved’ the fixed factor. Plot nested in study area was included as a random factor. The number of observations was N = 405.

Further, the effect of PUUV infection status on the mean minimum distance moved between recaptures (space use = consequence) was analysed with a GLMM with gamma distribution and a log link function (level of significance α < 5%) using R software. The dependent variable ‘mean minimum distance moved’ was transformed by adding 0.1 m to each value to eliminate zero values. In the initial model, PUUV infection status, sex, weight, interaction of sex as well as weight with PUUV infection status, and season were included as covariates. Plot nested in study area was included as a random factor. The number of observations was N = 405 and the best model was chosen according to AIC.

### Seroconversion and survival

Changes in PUUV infection status of recaptures (seroconversion) were analysed by Chi square tests (χ^2^). Analyses were performed using SPSS (*IBM SPSS Statistics for Windows, Version 22.0, 2013, IBM Corp., Armonk, New York*). Level of significance was α < 5%.

Survival of bank voles was analysed by a GLMM with binomial distribution and a logit link function (level of significance α < 5%) using R software (if necessary, post hoc tests were performed using ‘multcomp’ (Tukey contrasts) and ‘lsmeans’ (pairwise comparisons) packages). Survival (factorial variable; yes/no) was the dependent variable and PUUV infection status, sex, weight, interaction of sex as well as weight with PUUV infection status, and season were the initial fixed factors. Plot nested in study area was included as a random factor. The number of observations was N = 1263 and the best model was chosen according to AIC.

## Results

### Population density

**Fig. 1:**
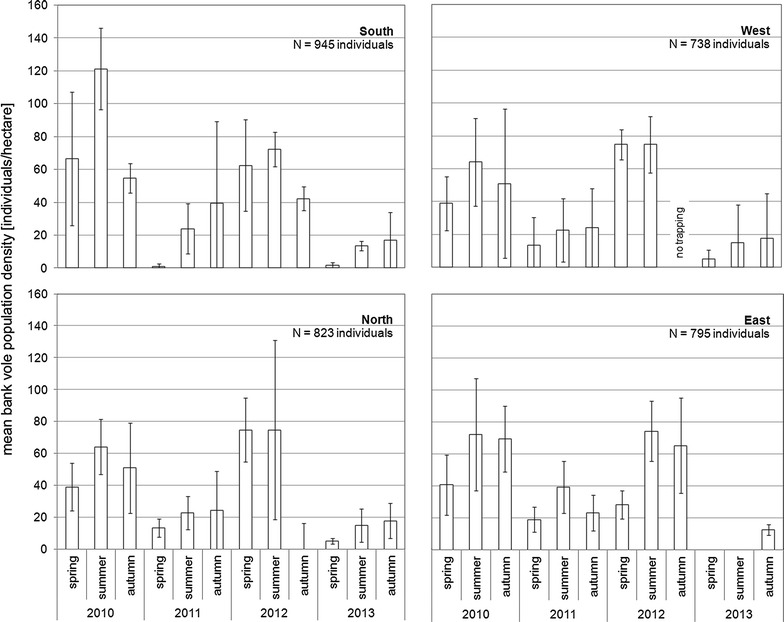
Population dynamics of bank voles in Germany from 2010 to 2013. Estimated mean population densities ± standard deviation as individuals per hectare from three replicate woodland plots per study area (N = total number of trapped bank voles)

In total 3301 bank voles were trapped during 2010–2013 which were roughly equally distributed among study areas. Population density tended to increase from spring to summer or autumn (Fig. 1). There were statistically significant differences in population densities between years (ANOVA: F = 34.54, *p* < 0.001) and seasons (F = 5.83, *p* = 0.004) but not between study areas (F = 0.50, *p* = 0.681). In 2010 and 2012, population densities were significantly higher than in 2011 and 2013 (Tukey’s HSD: 2010/2011 *p* < 0.001; 2010/2012 *p* = 0.933; 2010/2013 *p* < 0.001; 2011/2012 *p* < 0.001; 2011/2013 *p* = 0.043; 2012/2013 *p* < 0.001). Population density in summer was significantly higher compared to spring (*p* = 0.003) but not to autumn (*p* = 0.439) or between spring and autumn (*p* = 0.095). However, maximum densities were observed in summer of 2010 (61–121 ind/ha) and 2012 (72–82 ind/ha), respectively, indicating bank vole population outbreaks. The highest bank vole population density was estimated for summer 2010 in the South with on average 121 ind/ha (Fig. 1). In spring 2011 and 2013 population densities were lowest with 1–19 ind/ha (2011) and 0–5 ind/ha (2013).

### PUUV seroprevalence

2800 bank voles were tested for PUUV-reactive antibodies of which 566 (20%) were PUUV seropositive. Almost 99% (561 individuals) of those PUUV-seropositive bank voles were trapped in the South or West. In the North and East only 5 bank voles were tested positive in the PUUV IgG ELISA. Hence, PUUV is most likely not present at the trapping sites in North and East, which is in accordance with a large-scale study on bank voles from eastern Germany [15]. Drewes et al. [15] sampled about 1200 snap-trapped voles from multiple sites in Germany partly overlapping with the sites North and East from this study. All voles were PUUV-negative in IgG ELISA, conventional and real-time RT-PCR. Therefore, the positive test results from the two study areas reported here were most probably due to spillover infections of PUUV-related Tula virus (TULV) or false positive reactions in the ELISA. Because of the general absence of PUUV, North and East were not further included in data analyses regarding PUUV seroprevalence in bank voles.

**Fig. 2:**
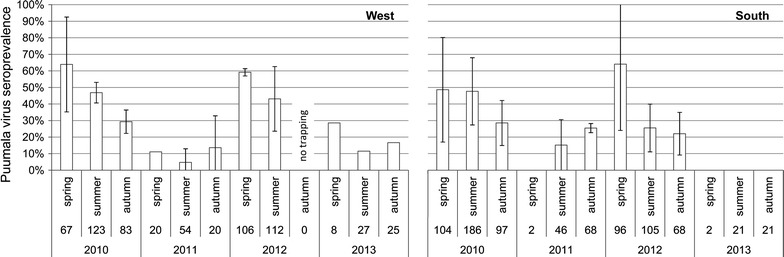
PUUV seroprevalence in bank vole populations in two regions in Germany from 2010 to 2013. Mean seroprevalence ± standard deviation (%) in spring, summer and autumn each year estimated from three replicate woodland plots per study area. Numerical values per season are total numbers of tested individuals of all plots in each study area

Serological investigations were performed for 1460 bank voles from West and South. Seroprevalence did not vary between study areas (ANOVA: F = 2.17, *p* = 0.145) but significantly differed between seasons (F = 4.35, *p* = 0.017) and years (F = 19.11, *p* < 0.001) (Fig. 2). Seroprevalences in 2010 and 2012 were significantly higher compared to 2011 and 2013 (Tukey’s HSD: 2010/2011 *p* **<** 0.001; 2010/2012 *p* = 0.998; 2010/2013 *p* < 0.001; 2011/2012 *p* < 0.001; 2011/2013 *p* = 0.367; 2012/2013 *p* < 0.001). Seroprevalence in spring was significantly higher compared to autumn (*p* = 0.018) but not to summer (*p* = 0.097) or between summer and autumn (*p* = 0.721). However, highest seroprevalence was found in spring 2010 (West: 64 ± 29%, South: 49 ± 32% per plot and trapping session) and 2012 (West: 59 ± 2%, South: 64 ± 40%). In these years, seroprevalence further decreased from spring to summer and from summer to autumn. In 2011 and 2013, the rate of PUUV-seropositive individuals in spring was much lower (<29%), and even zero, compared to the previous year. During those years, PUUV seroprevalence remained at a low or zero level or slightly increased towards autumn.

### Correlation of PUUV seroprevalence and bank vole population density

**Fig. 3:**
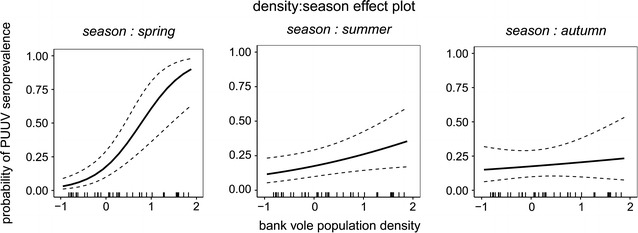
Seasonal effects of bank vole population density (z-transformed) on PUUV seroprevalence in the host population. *Black bars* on x-axis represent the distribution of population density values per trapping session

We performed a GLMM to test for the effect of bank vole population density on the variance in PUUV seroprevalence within populations. The higher the bank vole population density in a trapping session in spring the higher the PUUV seroprevalence (*p* < 0.001) (Fig. 3) (Table 1). A similar but weaker relation was found in summer (*p* = 0.027), but not in autumn (*p* = 0.570) (Table 1) (Fig. 3). There was no effect of the interaction of population density of the previous trapping session and season on PUUV seroprevalence (*p* > 0.05; removed from model). The random factor plot nested in study area (SA: plot) did not explain the variance in PUUV seroprevalence, but year did (0.31 ± 0.56; variance ± standard deviation). The best model explained almost one-third of the variance in PUUV seroprevalence (${\text{R}}_{\text{conditional}}^{2}$ = 0.28).

**Table 1:** Effects of bank vole population density in interaction with season on PUUV seroprevalence in the host population

Parameter	Estimate	(SE)	z	*p*
Intercept	−1.55	(0.34)	−4.61	*<0.001*
Bank vole population density:season ‘spring’	1.99	(0.44)	4.56	*<0.001*
Bank vole population density:season ‘summer’	0.51	(0.23)	2.21	*0.027*
Bank vole population density:season ‘autumn’	0.19	(0.34)	0.57	0.570

Random factor	Variance	(SD)		
SA: plot	0.00	(0.00)		
Year	0.31	(0.56)		

Parameter coefficients of generalized linear mixed model (GLMM) with binomial distribution

Number of observations = 58, degrees of freedom = 7

Italic values indicate significance of *p* value (*p* < 0.05)

### Space use

**Fig. 4:**
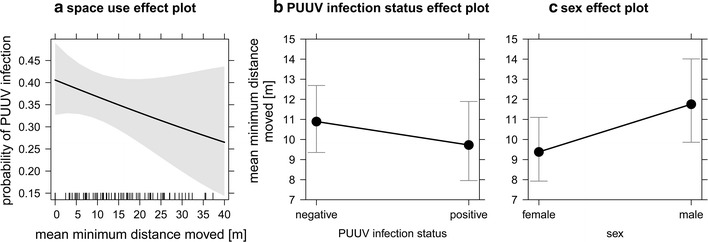
Effect plots of space use as potential cause (**a**) and consequence (**b**, **c**) of PUUV infections (and sex-c) in bank voles. Space use of bank voles = mean minimum distance moved. *Black bars* on x-axis (**a**) represent the distribution of individual ‘mean minimum distance moved’ values

Analysing the effect of space use as a cause for PUUV infections in bank voles using a GLMM showed no significant effect of the mean minimum distance moved on PUUV infection status (*p* = 0.205). The random factor plot nested in study area (SA: plot) could explain only a small portion of the variance in the probability of PUUV infection (0.01 ± 0.10; variance ± standard deviation) (Table 2; Fig. 4a).

**Table 2:** Model results of GLMMs investigating space use as cause (a) or consequence (b) of PUUV infections in bank voles

(a) Space use = cause				
Parameter	Estimate	(SE)	z	*p*
Intercept	−0.38	(0.17)	−2.20	*0.028*
Mean minimum distance moved	−0.02	(0.01)	−1.27	0.205

Random factor	Variance	(SD)		
SA: plot	0.01	(0.10)		

(b) Space use = consequence				
Parameter	Estimate	(SE)	t	*p*
Intercept	2.23	(0.38)	5.82	*<0.001*
PUUV infections status ‘positive’	−0.21	(0.13)	−1.55	0.120
Sex ‘male’	−0.90	(0.59)	−1.52	0.129

Random factor	*Variance*	*(SD)*		
SA: plot	0.00	(0.00)		

Parameter coefficients of generalized linear mixed models (GLMM) with binomial distribution (a) and gamma distribution (b)

Number of observations = 405, degrees of freedom = 3 (a) and 4 (b)

Space use = mean minimum distance moved between recaptures

Italic values indicate significance of *p* value (*p* < 0.05)

We also performed a GLMM to test for the effect of PUUV infection status on the mean minimum distance moved (space use = consequence). First of all, model selection excluded season, weight as well as all interactions from further consideration. No significant effect of PUUV infection status on space use was found (*p* = 0.378) (Table 2), although the mean minimum distance moved was 11.9% longer for individuals without PUUV-reactive antibodies (Fig. 4b). Males tended to move farther distances than females (*p* = 0.068) (Fig. 4c). The random factor plot nested in study area (SA: plot) could not explain the variance in the mean minimum distance moved.

### Seroconversion and survival

Most individuals trapped more than once were captured in two (122) and some in three (14) or four (2) trapping sessions. We recorded a total of 156 recaptures of individuals in multiple seasons. Usually, recaptures occurred in the following trapping session, but in two cases (one in each study area), voles first captured in summer were not recorded in autumn, but recaptured in the next spring. In both study areas, females were more frequently recaptured than males (West: 33/61 = 54%; South: 56/95 = 59%).

**Fig. 5:**
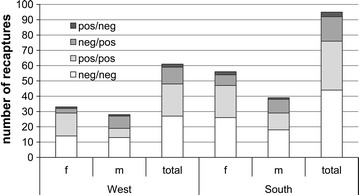
Number of recaptured bank voles. Recaptures per study area (West, South) in total and per sex (*f* female, *m* male) subdivided in recaptures that remained seronegative (neg/neg) or seropositive (pos/pos), seroconverted (neg/pos) or seemed to have lost antibodies (pos/neg)

Most recaptures remained seronegative (West 44%; South 46%) followed by bank voles that stayed seropositive (West 34%; South 34%) (Fig. 5). In the West 11 (18%) of recaptures indicated a PUUV seroconversion. In the South 16 voles (17%) seroconverted (Fig. 5). Two voles in the West (1%) and 3 voles in the South (2%) converted from seropositive to seronegative. More females than males remained seronegative (West: 14/27 = 52%; South: 26/44 = 59%) or seropositive (West: 15/21 = 71%; South: 21/32 = 66%). There was no sex difference in seroconversion (*p* > 0.05).

**Fig. 6:**
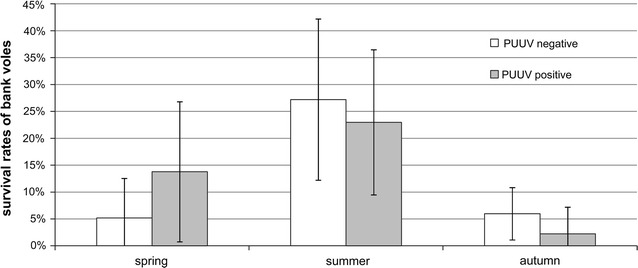
Survival rates of bank voles according to PUUV seroprevalence. Mean values ± standard deviation per season of first capture

Most recaptures occurred in 2010 and 2012 (78%). Seasonal variation in survival rates (Fig. 6) mirrored fluctuations in bank vole population density in outbreak years with peaks in summer (Fig. 1). A GLMM was performed to investigate the effect of PUUV infection status on survival of bank voles. First of all, the model showed significantly lower survival occurring from autumn to spring (over winter) in comparison to within year survival from spring to summer and summer to autumn (spring/summer z = −1.08, *p* = 0.520; spring/autumn z = −3.43, *p* = 0.002; summer/autumn z = −2.86, *p* = 0.011). This was most probably due to trapping intervals between seasons (twice as long over winter). Hence, separate GLMMs were performed for each season.

**Fig. 7:**
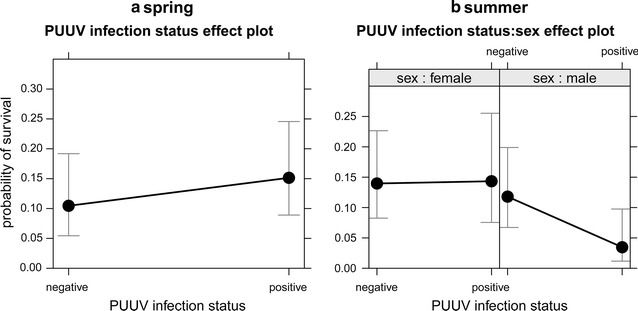
Effect of individual PUUV infection status on survival of bank voles from spring (**a**) to summer and from summer (**b**) to autumn. Significant results according to Table 3 are shown

Model selection excluded weight from further consideration in each season and all interactions in autumn (Table 3). From spring to summer, survival was significantly higher for PUUV-seropositive voles (*p* = 0.044) (Table 3; Fig. 7a) but did not vary between sexes. From summer to autumn, survival was significantly lower for seropositive (pos) males (M) in comparison to seropositive or seronegative (neg) females (F) (negF/posF z = −0.09, *p* = 1.000; negF/negM z = 0.67, *p* = 0.903; negF/posM z = 2.77, *p* = 0.027; posF/negM z = 0.63,

**Table 3:** Seasonal effects of PUUV infection status, sex and their interaction (not in c) on the survival of bank voles

(a) Spring				
Parameter	Estimate	(SE)	z	*p*
Intercept	−2.35	(0.45)	−5.21	*<0.001*
PUUV infections status ‘positive’	0.91	(0.45)	2.01	*0.044*
Sex ‘male’	0.42	(0.51)	0.82	0.414
PUUV infections status ‘positive’:sex ‘male’	−1.01	(0.63)	−1.59	0.112

Random factor	Variance	(SD)		
SA: plot	0.32	(0.56)		
Number of observations = 367, degrees of freedom = 5				

(b) Summer				
Parameter	Estimate	(SE)	z	*p*
Intercept	−1.82	(0.30)	−6.04	*<0.001*
PUUV infections status ‘positive’	0.03	(0.34)	0.09	0.930
Sex ‘male’	−0.19	(0.29)	−0.67	0.502
PUUV infections status ‘positive’:sex ‘male’	−1.35	(0.65)	−2.07	*0.038*

Random factor	Variance	(SD)		
SA: plot	0.25	(0.50)		
Number of observations = 599, degrees of freedom = 5				

(c) Autumn				
Parameter	Estimate	(SE)	z	*p*
Intercept	−2.86	(0.37)	−7.75	*<0.001*
PUUV infections status ‘positive’	−1.07	(0.76)	−1.41	0.160
Sex ‘male’	0.87	(0.47)	1.87	0.062

Random factor	Variance	(SD)	z	*p*
SA: plot	0.00	(0.00)		
Number of observations = 297, degrees of freedom = 4				

Parameter coefficients of generalized linear mixed models (GLMM) with binomial distribution

Italic values indicate significance of *p* value (*p* < 0.05)

*p* = 0.919; posF/posM z = 2.64, *p* = 0.038; negM/posM z = 2.38, *p* = 0.076) (Table 3; Fig. 7b). Over winter (autumn to spring), no effect of PUUV infection status or sex on survival was found (Table 3).

## Discussion

The processes involved in hantavirus transmission have been mostly studied in Northern Europe [26, 27, 37, 38]. Knowledge about mechanisms in Central Europe is sparse [17, 21, 39–43]. With our recent study, we covered two spatially replicated bank vole outbreaks (2010 and 2012) and two low phases (2011 and 2013), which is likely to provide robust estimation of relevant patterns and processes.

Population dynamics of bank voles in Central Europe are driven by seed masting of beech trees [18–20, 22, 23]. As beech mast events have recently occurred every 2–3 years [17, 20, 22], bank vole outbreaks have also occured every 2–3 years. This 2–3 year cycle has a major effect on PUUV dynamics within the rodent host population and hence on the number of human PUUV infections.

In our study, PUUV seroprevalence (reliably detected only in two regions) temporally fluctuated depending on host population abundance. Highest seroprevalences were found in 2010 and 2012, when rodent host abundance peaked (Fig. 1, upper graphs) triggered by beech mast events in 2009 and 2011 [22], and coincided with the highest numbers of human PUUV infections ever recorded since the disease became notifiable in 2001 (SurvStat@RKI 2.0, https://survstat.rki.de, data status: 17.05.2016). In bank vole outbreak years, PUUV seroprevalence peaked in spring, when populations consisted of old overwintered animals [37, 44]. These animals most probably die by summer, leading in part to the decrease in PUUV seroprevalence, although population density increases [37, 44]. Further, despite increasing absolute numbers of infected individuals, PUUV seroprevalence decreases (Fig. 2) indicating that population growth rate outperforms transmission rate in peak reproductive phase. Hence, uninfected young of the year lower PUUV seroprevalence during the year [45, 46] (see below). Accordingly, this decrease in PUUV seroprevalence was observed to proceed until autumn. In 2011 and 2013, when bank vole densities collapsed in the West and South, seroprevalence also drastically decreased.

Density-dependence of virus occurrence in the rodent host population was demonstrated for hantavirus [45–47]. However, we found also clear differences in PUUV seroprevalence among seasons. There was a strong direct density-dependence in spring. This might be due to the presence of many overwintered individuals that contracted PUUV in the previous year or during winter and represent the founder population for the upcoming outbreak, while in years with smaller spring populations (not leading to an outbreak), PUUV seroprevalence was much lower. In summer, when uninfected newborns without maternal antibodies (for details see Kallio et al. [48]) enter the population, PUUV seroprevalence is lowered (the juvenile dilution effect—[46]) and consequently density-dependence was decreased, but still significant. In autumn, there was no density effect and density-dependence seems to be diluted over the course of the year likely due to a more complex transmission scenario during the reproduction phase based on reproductive behaviour (e.g. aggression, territoriality; [49]). Our data underlines the dependence of PUUV seroprevalence on seasonal and multi-annual density dynamics of the rodent host in Central-Europe [37, 47].

Considerable temporal variation and the geographic differences in host and virus dynamics were detected indicating that the PUUV-bank vole-human epidemiological system is even more complex than previously assumed. Perhaps most strikingly, PUUV is virtually absent from the German east and north although bank voles are present [15] and fluctuations in abundance are also related to beech masting [22]. Bank voles in the North and East belong to the Eastern and Carpathian evolutionary lineages, whereas bank voles in the South and West belong to the Western evolutionary lineage [15]. The almost complete absence of PUUV-seroreactive bank voles in the current study from sites in North and East is in line with results of a previous study where bank voles from northern and eastern parts of Germany were found to be free of PUUV as determined by IgG-ELISA and RT-PCR analyses [15]. The PUUV absence in the eastern and northern part of Germany is caused by the association of PUUV with the Western evolutionary lineage of the bank vole and its distribution following the postglacial recolonization of Germany [15]. The few PUUV-seroreactive bank voles found in the current study are probably the result of spillover infections, most likely by TULV. TULV is the only hantavirus present in other vole species which is antigenically closely related to PUUV and PUUV- and TULV-specific antibodies cannot be differentiated by ELISA [50, 51]. However, spillover infections seem to occur only rarely (Drewes et al. unpublished data) [2, 30] and hence did not affect the present results.

Male bank voles move farther than female bank voles [39, 52], which could not be confirmed statistically in our study, although males seemed to be more mobile (25.4% farther mean minimum distance moved). More active males are more prone to infection [44, 53], which could lead to the hypothesis that PUUV seroprevalence is positively correlated with space use causing an increased infection probability. Nevertheless, no effect of space use as a cause for PUUV infections in bank voles could be found. A reverse connection of both parameters (reciprocal effect; [32]) might indicate a possible sublethal effect on rodent host fitness (here presented by space use) as a consequence of PUUV infection. However, an effect of PUUV infection status on space use could not be detected, which contrasts with earlier findings that PUUV infection affects host fitness with regards to survival and reproduction [42, 54]. In general, shorter mean minimum distances moved seem to be associated with a higher probability of PUUV infection in bank voles (Fig. 4a, b), but it remains unclear whether PUUV infections affects spatial activity or vice versa. The individual movement data generated by recapture studies are rather crude and more detailed data from telemetry work is needed to further elucidate cause as well as consequences of PUUV infection on rodent host fitness and behaviour.

The proportion of PUUV-seronegative and -seropositive voles or voles with a seroconversion corresponded to earlier findings [39, 55]. Seroconversion in our study (17–18%) was smaller compared to former results (32%; [39]). This is most probably an effect of trapping intervals, which were mostly three months in our study and 6 months in Escutenaire et al. [39], decreasing the probability of seroconversion in our study. ‘Inverted seroconversion’ from positive to negative in three juveniles (first capture ≤15 g) was most likely a consequence of loss of maternal antibodies [56]. Although the presence of PUUV-reactive antibodies is assumed to persist lifelong [57], the loss of antibodies in recaptured animals might indicate a virus clearance [58], as discussed also for another hantavirus [59], or might be caused by an oscillation of the antibody titer below the detection limit of the ELISA used.

Females were more frequently recaptured than males (male:female = 1:1.3). Total capture of females was also higher than males (male:female = 1:1.2) and can be explained by stronger territoriality of females than males [60]. Seroconversion did not differ between sexes. Hence, general assumptions that males have a higher infection risk due to their behavior [44, 47, 53] or that females probably better survive with subject to PUUV infection [54] were not supported. Thus, supposed effects of PUUV on vole fitness [54] require further investigations.

Recaptures between trapping sessions reflecting survival were more frequent in outbreak years when the initial population abundance was higher. Survival also followed seasonal patterns because there was higher survival at the end of the reproductive phase (summer to autumn) for PUUV seronegative and seropositive captures. Over-winter survival was lowest, which is most likely an effect of trapping interval (over winter twice as long as within year) and hence survival according to PUUV infection status was analysed separately for each season. PUUV-seropositive voles survived better from spring to summer, which might indicate a positive effect on host fitness. However, an alternative plausible explanation is the effect of residency. In spring, especially in outbreak years, PUUV seroprevalence is increased reflected by more seropositive than seronegative voles. PUUV-seropositive animals that survived are most probably local residents, which got infected in the previous year and overwintered. Residents are more likely to be recaptured leading to more recapture-based survival than in seronegatives. The latter may include transient or immigrating voles that are recaptured less often than residents. From summer to autumn, PUUV-seropositive males survived less compared to females of either infection status. Again, this might have been an effect of residency. In summer, PUUV seroprevalence is decreased and proportionally more individuals are seronegative most probably due to uninfected young voles. Not only residents but also females (territoriality; [60]) are more likely to be recaptured. Accordingly, males are more likely to disperse, which could have led to less recaptures (apparent reduced survival). Over winter, no effect of PUUV infection on survival was found. Seasonal survival has not been explored so far. Former studies investigating over-winter survival report no effect of PUUV infection on survival [44, 61], but see Kallio et al. [54]. Maternal antibodies did not matter in our study because the number of juvenile recaptured with potential maternal antibodies was negligible (spring to summer N = 1, summer to autumn N = 4). Little is known about sublethal effects of hantavirus infection on behaviour and fitness of rodent hosts that may have consequences for survival and population dynamics. Clarifying such impacts could shed light on our inconsistent findings about the relation of PUUV infection status on survival such as apparent increased survival of PUUV seropositives from spring to summer (maybe induced by an increased PUUV seroprevalence in spring) and apparent decreased survival of seropositive males from summer to autumn. Therefore, further research—preferably in controlled environments—is warranted.

## Conclusion

Our study revealed relevant patterns and processes in the dynamics of PUUV and its rodent host in Central Europe. Seasonal and multi-annual fluctuations of PUUV seroprevalence depend on host abundance. This knowledge can facilitate the future development of early warning systems to lower the risk of human hantavirus infections.

Effects of PUUV infection on rodent host space use and survival could not be conclusively clarified. Further research is required to test for such impacts possibly by comparing behaviour and survival between endemic and non-endemic regions.
